# Cerebral Oximetry in Syncope and Syndromes of Orthostatic Intolerance

**DOI:** 10.3389/fcvm.2019.00171

**Published:** 2019-11-22

**Authors:** Isabella Kharraziha, Hannes Holm, Erasmus Bachus, Fabrizio Ricci, Richard Sutton, Artur Fedorowski, Viktor Hamrefors

**Affiliations:** ^1^Department of Clinical Sciences, Lund University, Malmö, Sweden; ^2^Department of Internal Medicine, Skåne University Hospital, Malmö, Sweden; ^3^Department of Cardiology, Skåne University Hospital, Malmö, Sweden; ^4^Institute of Cardiology, University “G. d'Annunzio”, Chieti, Italy; ^5^Department of Neuroscience and Imaging, ITAB - Institute Advanced Biomedical Technologies, University “G. d'Annunzio”, Chieti, Italy; ^6^National Heart and Lung Institute, Imperial College, Hammersmith Hospital, London, United Kingdom

**Keywords:** syncope, vasovagal syncope, cerebral oxygenation, hemodynamics, postural orthostatic tachycardia syndrome, head-up tilt

## Abstract

Cerebral autoregulation is crucial for maintaining cerebral blood flow and perfusion. In recent years, the importance of cerebral oxygenation in syncope and orthostatic intolerance (OI) has received increased attention. Cerebral tissue oxygenation can be measured by using near-infrared spectroscopy (NIRS), which determines the ratio of oxygenated hemoglobin to total hemoglobin in cerebral tissue. NIRS is non-invasive technology using near-infrared light, which displays real-time cerebral tissue oxygenation. Normal values of cerebral tissue oxygenation in healthy subjects are 60 to 80%. Head-up tilt test (HUT) offers the opportunity to observe the haemodynamic changes precipitating syncope and is, today, the standard method for the evaluation of syncope and orthostatic intolerance syndromes. In previous studies where NIRS was applied during HUT, a significant decrease in cerebral tissue oxygenation both prior to and during loss-of-consciousness in vasovagal syncope (VVS) has been observed. Interestingly, cerebral tissue oxygenation appears to decrease even before haemodynamic changes can be observed. Apart from VVS, cerebral tissue oxygenation decreases during orthostatic provocation in patients with orthostatic hypotension (OH) and postural orthostatic tachycardia syndrome (POTS), in the latter even in the absence of hypotension. Importantly, decline of cerebral tissue oxygenation in VVS and POTS during HUT may not correlate with hemodynamic changes. In this mini review, we summarize the current knowledge of the application of cerebral oximetry in syncope and orthostatic intolerance syndromes, discuss its likely value as a clinical diagnostic tool and also emphasize its potential in the understanding of the relevant pathophysiology.

## Introduction

Syncope and orthostatic intolerance (OI) involve a variety of clinical syndromes on the basis of cardiovascular disease or autonomic dysfunction (1). These clinical entities range from benign sporadic episodes, such as vasovagal syncope (VVS), to severely disabling symptoms of autonomic failure in advanced forms of orthostatic hypotension (OH) (1, 2). Syncope and OI are often provoked by orthostatic stress accompanied by debilitating symptoms of cerebral hypoperfusion (3). Although, a number of methods, including long-term ECG-monitoring, head-up-tilt-test (HUT) with continuous haemodynamic measurements and other specific cardiovascular autonomic tests are available, the identification of triggers for syncope and OI in a specific case may create a challenge for a clinician (1). In such setting, the measurement of cerebral tissue oxygenation may add important diagnostic information and therapeutic clues. In this mini review, we summarize the current knowledge of the application of cerebral oximetry in syncope and OI, discuss a possible role in diagnosis and its value in understanding the pathophysiology of VVS and syndromes of OI.

## The Technical Background of Cerebral Tissue Oxygenation Measurement

**Figure 1:**
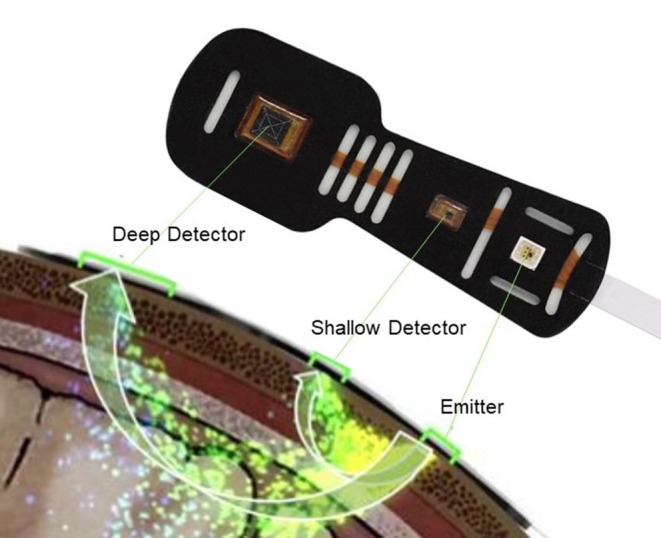
Measurement of cerebral tissue oxygenation with near infrared spectroscopy. The NIRS probe is attached to the forehead. Near infrared light from the emitter, passes through tissues such as skin and bone with minimal absorption whereas hemoglobin (Hb) has a well-defined absorption spectrum that is influenced by the binding of O_2_. Light attenuation is detected by the deep and shallow detectors as shown in this figure. Because oxygenated Hb and deoxygenated Hb have different absorption spectra, their proportion can be calculated. In healthy human normal cerebral tissue oxygenation is 60–80%. Edwards Lifesciences Corp, Irvine, CA, USA images archive.

There are several methods that can be used in measuring the cerebral circulation, including ultrasound-based techniques such as transcranial doppler. In recent years, the use of near infrared spectroscopy (NIRS) has gained increased attention since it offers the opportunity to observe absolute values of cerebral oxygenation (4). NIRS measures the ratio between oxygenated hemoglobin and total hemoglobin (Hb) which reflects a proportional mix of arterial and venous blood in the outer regions of the brain (5). Near infrared light (700–1,000 nm) passes through tissues such as skin and bone with minimal absorption whereas hemoglobin (Hb) has a well-defined absorption spectrum that is influenced by the binding of O_2_. Because oxygenated Hb and deoxygenated Hb have different absorption spectra, their proportion can be calculated (5). The principles of NIRS methodology in the measurement of cerebral tissue oxygenation are shown in Figure 1. In a population of healthy individuals, normal cerebral tissue oxygenation has been established to be between 60 and 80% (6).

NIRS technology combined with so called diffuse correlation spectroscopy permits direct monitoring of cerebral blood flow in addition to oxygenation (7). In short, the diffuse correlation spectroscopy flow-oximeter uses near infrared light to detect motion of moving scatters, primarily red blood cells, to directly and non-invasively measure local cerebral blood flow (7). This method is less commonly used in the field of autonomic dysfunction but may emerge as a potent assessment in the future. Absolute cerebral blood flow can also be measured with techniques such as functional magnetic resonance imaging and positron emission tomography. In addition, absolute measurements of cerebral blood flow using NIRS have been performed with tracer tracking techniques. The two main tracers have been oxyhemoglobin and indocyanine green (4).

## Monitoring of Cerebral Oximetry in Syncope Patients

Head-up tilt test (HUT) is an established method for the evaluation of unexplained syncope and orthostatic intolerance by its opportunity to observe the haemodynamic changes during orthostatic provocation (8). By applying cerebral oximetry during orthostatic provocation by HUT it can be observed that cerebral tissue oxygenation decreases also in normal subjects, even though the decrease is small (9–11). In contrast, a more pronounced decrease in cerebral tissue oxygenation has been observed during HUT in patients with syncope and symptoms of orthostatic intolerance. The magnitude of this decrease is dependent both on the underlying diagnosis as well as the timing of the measurement in relation to the time of pre-/syncope (9, 12–15). Vasovagal syncope (VVS) is the most common cause of syncope (16). The underlying pathophysiological mechanisms of VVS are not fully understood but it has been proposed that reduction in cardiac output rather than reduction in systemic vascular resistance is the main cause of hypotension in VVS (17). The reduction in cardiac output may affect cerebral perfusion by increased cerebral vascular resistance, indicating that cerebral perfusion is dependent on a sufficient cardiac output and arterial inflow pressure (18). Thus, it is also likely that cerebral autoregulation, which aims to maintain constant cerebral blood flow over a mean arterial blood pressure (MAP) range of 50–150 mmHg (19), has a significant role in VVS pathophysiology.

**Figure 2:**
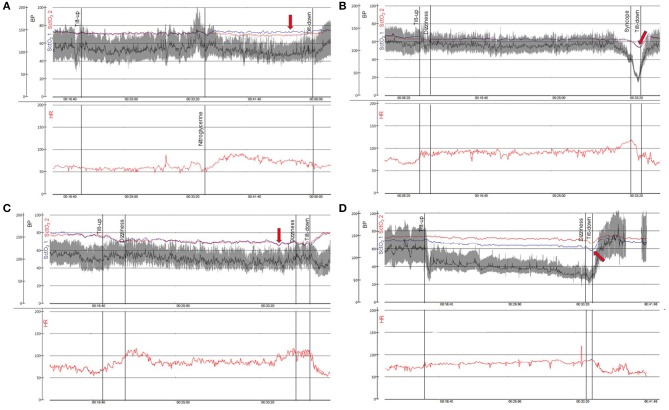
Cerebral oximetry during head-up tilt in patients with syncope and orthostatic intolerance. In **(A–D)** two panels are shown: blood pressure in gray/black (BP—mmHg−0–200) and cerebral tissue oxygen oxygenation in blue and red (SctO_2_-%−0–100) are plotted above with time scale denoted. Heart rate shown in red (HR—bpm−0–200) is demonstrated simultaneously in the panel below. **(A)** 61-year-old woman with syncope while driving. There is a normal response to head up tilt and preserved cerebral tissue oxygenation (red arrow). **(B)** 20-year-old woman with history of recurrent syncope. Vasovagal syncope during head up tilt occurs when cerebral tissue oxygenation falls below 60% (red arrow). **(C)** 23-year-old man with orthostatic intolerance. This patient fulfills diagnostic criteria of postural orthostatic tachycardia syndrome with pronounced decrease in cerebral tissue oxygenation during head up tilt of more than 10% associated with subjective symptoms (dizziness, red arrow). **(D)** 77-year-old man with Parkinson's disease and orthostatic hypotension. The symptoms of cerebral hypoperfusion are intolerable when cerebral tissue oxygenation falls below 60% (red arrow). BP, blood pressure; SctO2, cerebral tissue oxygenation; HR, heart rate. SctO2 1 measures the left side of the frontal lobe and SctO2 2 measures the right side of the frontal lobe.

When standing up, venous blood is pooled in the lower extremities and splanchnic area which leads to decreased venous return and reduced cardiac output. In turn, decreased cardiac output initiates various autonomic responses that if insufficient or impaired, ultimately may result in decreased cerebral perfusion and syncope (18). The implementation of cerebral oximetry in syncope patients has led to several important discoveries, including the ability to predict the onset of VVS before obvious changes in haemodynamic parameters are manifest by showing a gradual decrease in cerebral oxygenation prior to syncope (9, 11). The Bachus et al. (9) study reported a significant decrease in cerebral tissue oxygenation 1 min prior to reflex activation in contrast to MAP which did not show a significant decrease at this time. Another interesting finding from the same study was that syncope occurred when cerebral tissue oxygenation had fallen below 60% (9) (Figure 2). Hence, the fall in cerebral tissue oxygenation may explain the discrepancy between symptoms reported by the patient and haemodynamic parameters, for instance, in the prodrome of VVS or unexplained symptoms of orthostatic intolerance.

Another study using diffuse correlation spectroscopy flow-oximetry found that a threshold of ~50% cerebral blood flow decrease during HUT can be applied in order to separate pre-syncopal patients from controls. Potentially, continuous monitoring of cerebral tissue oxygenation and blood flow may provide predictive information for impending VVS during HUT, possibly both shortening the test and avoiding the need for induction of syncope (20, 21).

Cerebral oximetry may also offer the possibility to analyse the syncope mechanism in potentially overlapping diagnoses such as VVS, postural orthostatic tachycardia syndrome (POTS), or OH by providing characteristic signal patterns during HUT (9, 11) (Figure 2). Different patterns of cerebral tissue oxygenation may aid in distinguishing between autonomic failure and reflex activation. In a previous study using diffuse correlation spectroscopy flow-oximetry, two stages of physiological responses were observed in pre-syncopal patients during HUT including gradual changes in cerebral blood flow during Stage I followed by rapid and dramatic cerebral blood flow changes during Stage II (20) associated with failure of physiological autoregulation. This two-stage pattern, characteristic of VVS, has also been reported in a study using NIRS only (9).

## Monitoring of Cerebral Oximetry in Orthostatic Intolerance and Pots

An issue with HUT is the apparent high false-positive and false-negative rates in adults (22) although alternative explanations have been provided (23). By adding NIRS during HUT, clinicians can detect early changes in cerebral oxygenation and thereby signs of cerebral hypoperfusion. During orthostatic provocation, cerebral tissue oxygenation decreases even in healthy patients (24, 25) but to a lesser degree. Patterns are relatively easy to detect when monitored in association with hemodynamics.

Kim et al. (11) using cerebral oximetry, found that total cerebral Hb (oxygenated Hb and deoxygenated Hb) after tilt-up decreases temporally during orthostasis, even in healthy controls. However, compared with healthy controls, the recovery of Hb concentration to baseline values seems to occur later in patients with OI, even in those who experience a normal response to HUT. The point at which the total Hb concentration recovered was 200 s after tilt-up in controls in contrast to patients with orthostatic intolerance where the blood volume continued to fall for ~450 s. This delay in total Hb recovery may indicate a delay in return to cerebral homeostasis compared with healthy controls and perhaps impaired cerebral autoregulation. Hence, by adding cerebral oximetry during HUT, cerebral effects of orthostatic intolerance can be detected (11). This finding may also have relevance to that of McCrory et al. who found that the speed of recovery of blood pressure after active stand in community dwelling older adults offered valuable prognostic information concerning aging and mortality (26).

Cerebral tissue oxygenation declines during orthostasis in patients with POTS (10, 15) (Figure 2C). POTS is a disorder predominately found in subjects <40 years and is characterized by orthostatic intolerance and heart rate increase ≥30 bpm during orthostasis in the absence of OH. In addition to reduced orthostatic tolerance, patients with POTS frequently experience debilitating symptoms such as light-headedness, nausea, blurred vision, fatigue, mental confusion (“brain-fog”), chest pain and gastrointestinal problems (27). Syncope may occur in some patients although pre-syncopal symptoms are more common (28). Light-headedness and cognitive deficits are among the most disabling symptoms in POTS and have been assumed to be a result of cerebral hypoperfusion despite normal blood pressure (29). Several studies have measured changes in cerebral blood flow velocity with transcranial doppler in POTS patients during active standing test or HUT, but the findings are inconsistent (15, 29–32). However, it has recently been demonstrated that POTS patients experience lower cerebral tissue oxygenation during HUT compared with HUT negative patients (10). Interestingly, the decrease in cerebral tissue oxygenation in POTS correlated weakly with heart rate increase, implying that other factors may be responsible for the lower cerebral tissue oxygenation (10). Another study using both NIRS and transcranial doppler on POTS patients revealed a decrease in cerebral oxygenated Hb during HUT but no significant decrease in cerebral blood flow velocity compared with controls (15). In conclusion, cerebral oximetry appears to detect a dysfunctional response to orthostatic provocation where transcranial Doppler does not. The lower cerebral tissue oxygenation detected in POTS patients prompts a hypothesis of disrupted homeostasis of cerebral oxygenation. However, it is not known whether lower cerebral tissue oxygenation in POTS patients is a consequence of or a partial cause of POTS symptoms.

## Advantages and Limitations of Cerebral Oximetry

NIRS and diffuse correlation spectroscopy flow-oximetry have definite potential for practical measurements of cerebral perfusion. They are both safe, non-invasive, applicable at the bedside, and suitable for quantitative measurements and continuous monitoring with little operator-dependency. Wireless NIRS devices have recently been validated in animals (33) and are soon anticipated to be approved for human use. Other non-invasive techniques that monitor cerebral blood flow, including magnetic resonance imaging, positron emission tomography and single photon emission computed tomography are all limited by availability, cost and the difficulty of making continuous bedside measurements. These imaging techniques also involve ionizing radiation. Transcranial doppler is operator-dependent and has wide inter-examiner variability (4).

The limitations of cerebral oximetry are mainly technical. NIRS does not measure cerebral blood flow directly, unless some form of flow tracer is used, e.g., a short breath of 100% oxygen or an injection of a contrast agent (4). As previously mentioned, diffuse correlation spectroscopy flow-oximetry does not have this problem. Another limitation of NIRS is the risk of measuring the saturation of overlying tissues or deeper regions of the brain (34). Although changes in cerebral tissue oxygenation have been correlated with circulatory responses induced by orthostatic blood pressure changes (9, 13, 25), the skin blood flow shift from head to lower body induced by orthostasis could also affect NIRS measurements. The risk of measuring superficial tissue can be diminished by adding detectors at multiple distances from the emitted light source. Other factors that can affect the signal are motion artifacts, melanin pigmentation in hair and bilirubin in patients with jaundice. Melanin content in skin does not affect the results as it is limited to the superficial part of the skin (34).

Further, NIRS displays a great variety in the human anatomy of vessels and non-vascular tissue, where baseline values vary by ~10% between individuals. Thus, cerebral oximetry is more suitable for detecting intra-individual rather than inter-individual changes (34). Also, there is an issue of reproducibility in cerebral oximetry. However, according to a small study (24), deoxygenated Hb measured by NIRS seems to be reproducible and may therefore be used in follow-up studies.

Finally, cerebral oximetry with NIRS measures oxygen saturation levels in the regions where the probes are located and does not provide information on cerebral saturation in remote parts of the brain. A significant redistribution of the cerebral blood flow values during HUT has previously been found (35), with a reduction in frontal and an increase in postcentral areas. This frontal flow decrease was greater in OH patients than in healthy controls. However, another study found the reduction of brain perfusion during orthostatic stress to be global (36), which may indicate that the issue of redistribution is not of great significance provided that the scalp electrodes are placed on the same regions. In addition, previous studies have found NIRS to be comparable with functional magnetic resonance imaging and positron emission tomography which both measure changes in cerebral blood flow globally (37, 38).

## Future Applications of Cerebral Oximetry in Syncope and Orthostatic Intolerance

As described in this review, cerebral oximetry has been increasingly used in various experimental settings in the recent years. However, cerebral oximetry is yet to find its role as an established method in the clinical settings of syncope and orthostatic intolerance. The future applications of cerebral oximetry can be broadly divided into two partly overlapping major goals: First, cerebral oximetry may aid in the understanding of pathophysiological mechanisms underlying syncope and orthostatic intolerance, the relationship between haemodynamic changes and cerebral autoregulation, and also serving as a complement to the current methods of cerebral circulation assessment. The cerebral oximetry may be applied for better monitoring of coupling between haemodynamic changes and characteristic symptoms observed in both VVS (9, 11, 20) and the complex syndromes of orthostatic intolerance, such as POTS (10, 15). Second, cerebral oximetry may aid in the clinical diagnosis and possibly in tailoring therapy against syncope and orthostatic intolerance.

As an example, monitoring of cerebral tissue oxygenation may provide predictive information for impending VVS during HUT, avoiding the need for induction of syncope (20, 21). The treatment implications conferred by cerebral oximetry are highly related to the ability of the method to enhance our understanding of the underlying mechanisms of syncope and orthostatic intolerance. However, since it is likely that there exists inter-individual threshold for when low cerebral tissue oxygenation causes symptoms, it cannot be excluded that cerebral oximetry in itself may also prove useful for targeting preventive therapy in syncope and orthostatic intolerance, despite this reasoning being highly speculative at this stage.

## Conclusion

Cerebral oximetry with near infrared spectroscopy is a harmless, non-invasive technique which provides the opportunity to measure real-time regional cerebral tissue oxygenation. With the addition of diffuse correlation spectroscopy, simultaneous estimation of cerebral blood flow can be made. These techniques are able to demonstrate different patterns of cerebral deoxygenation during orthostatic provocation in patients with various types of syncope and orthostatic intolerance, before any haemodynamic changes can be observed. Furthermore, the addition of cerebral oximetry to the established head up tilt test allows a more sensitive detection of orthostatic intolerance and may reflect disrupted homeostasis of cerebral oxygenation in POTS. With increasing use in research, cerebral oximetry may be able to provide information about factors related to cerebral hypoperfusion as well as clues to hitherto unknown factors behind syndromes associated with recurrent syncope and orthostatic intolerance.

## Author Contributions

Concept and design, drafting the article, critical revision of the article, and approval of the article: IK, HH, EB, FR, RS, AF, and VH. Funding secured by: AF and VH. Other: IK and AF.

### Conflict of Interest

AF reports personal fees from Medtronic Inc. and Cardiome Corp., and patent royalties from Thermo Fisher Scientific outside the submitted work. RS reports consultancy for Medtronic Inc., Member of Speakers' Bureau Abbott Labs and Stockholder in Edwards LifeSciences, Boston Scientific Inc. and AstraZeneca PLC. VH reports previous educational congress grant from Boston Scientific Inc. outside the submitted work and Stockholder in Swedish Orphan Biovitrum AB (publ). EB has been employed by AstraZeneca after completion of this study. The remaining authors declare that the research was conducted in the absence of any commercial or financial relationships that could be construed as a potential conflict of interest.
